# Pain sensing neurons promote tissue regeneration in adult mice

**DOI:** 10.1038/s41536-021-00175-7

**Published:** 2021-10-14

**Authors:** Lise Rabiller, Elodie Labit, Christophe Guissard, Silveric Gilardi, Bruno P. Guiard, Lionel Moulédous, Marine Silva, Gilles Mithieux, Luc Pénicaud, Anne Lorsignol, Louis Casteilla, Cécile Dromard

**Affiliations:** 1 RESTORE, UMR INSERM 1301/CNRS 5070/Université Paul Sabatier/EFS/ENVT, Toulouse, France; 2 Centre de Recherches sur la Cognition Animale (CRCA), Centre de Biologie Intégrative (CBI), Université de Toulouse, CNRS UMR-5169, UPS, Toulouse, France; 3 INSERM U1213, Université Lyon 1, Lyon, France; 4 Present Address: Department of Physiology and Cell Information Systems, McGill University, Montreal, QC Canada; 5 Present Address: Alan Edwards Center for Research on Pain, McGill University, Montreal, QC Canada; 6 Present Address: Department of Comparative Biology and Experimental Medicine, Faculty of Veterinary Medicine, University of Calgary, Calgary, AB Canada

**Keywords:** Peripheral nervous system, Homeostasis

## Abstract

Tissue repair after injury in adult mammals, usually results in scarring and loss of function in contrast to lower vertebrates such as the newt and zebrafish that regenerate. Understanding the regulatory processes that guide the outcome of tissue repair is therefore a concerning challenge for regenerative medicine. In multiple regenerative animal species, the nerve dependence of regeneration is well established, but the nature of the innervation required for tissue regeneration remains largely undefined. Using our model of induced adipose tissue regeneration in adult mice, we demonstrate here that nociceptive nerves promote regeneration and their removal impairs tissue regeneration. We also show that blocking the receptor for the nociceptive neuropeptide calcitonin gene-related peptide (CGRP) inhibits regeneration, whereas CGRP administration induces regeneration. These findings reveal that peptidergic nociceptive neurons are required for adult mice tissue regeneration.

## Introduction

Despite clinical advances, adult humans cannot regenerate injured tissue after traumatic lesion or surgical intervention. Instead, tissue trauma repair leads to extracellular matrix accumulation and ultimately to the formation of a scar, fibrosis and to loss of function^1^. How some organisms such as newt and zebrafish regenerate lost tissue is largely investigated and important features of the regenerative process have been identified using such model organisms. Among the factors required for successful regeneration, innervation comes forward as a crucial parameter. Nearly every known example of vertebrate tissue regeneration ranging from fish to mammal requires the presence of intact peripheral nerves. The amputated pectoral fin of zebrafish^2^ as well as the limb of the salamander^3,4^, the ear of the MRL-MPJ mouse^5^, and the fingertip of the C57Bl/6 mouse^6^ can no longer regenerate if it has undergone denervation. However, these studies are based on neurotomy, which does not distinguish the contribution of autonomous nerve fibres from that of sensory ones.

Sensory nerves provide information on the presence of a striking diversity of stimuli; but within peripheral damaged tissue, noxious stimuli are transduced by specialized pain sensing neurons, which are primary nociceptive sensory neurons, responsible for the first stage of pain sensations. Their cell body resides within the dorsal root ganglia and their axon bifurcates, creating one branch projecting to the periphery where specialized free endings course throughout the peripheral tissues and another branch projecting into the central nervous system^7^. When activated, they release neuropeptides such as calcitonin gene-related peptide (CGRP), which is one of the most relevant neuropeptides in the transmission of nociceptive signals. Their function can be modulated by endogenous opioids released from immune cells^8^ or by administration of opioid drugs. The analgesic effect of morphine-like opioids is mainly mediated by µ-opioid receptors (MOR) and is carried out through the inhibition of sensory neuropeptides released from the central and peripheral endings of the nociceptive neurons^9,10^.

To determine whether nociceptive neurons are involved in tissue regeneration, we used our model of massive resection of the subcutaneous adipose tissue (scAT) in adult mice. ScAT is a complex tissue that displays high plasticity in adults. Because of its central role in energy homeostasis, it can undergo phenotypic and size modifications depending on the metabolic context^11,12^. Its location just under the skin makes it easily accessible and its specific anatomy allows a good reproducibility of the resection. ScAT is therefore a relevant model for studying the plasticity of organs post-lesion in adult mammals. Using this model, we have recently shown that peripheral opioids inhibit regenerative ability in adult vertebrates including mammals^13^.

In the present study, we first sought to establish the involvement of MOR in the deleterious effect of endogenous opioids on regenerative process by evaluating the nociception and the regenerative capacity of mice disabled for this receptor. Given that MOR are expressed primarily in peripheral nociceptive neurons, we examined the requirement for nociceptive neurons in the regenerative process. Then, we explored the contribution of the CGRP neuropeptide to the nerve-dependent regeneration and we evaluated if co-administration of CGRP with morphine could induce tissue regeneration while allowing analgesia. Altogether, our findings reveal that nociceptive nerves promote tissue regeneration likely through the peripheral release of the CGRP neuropeptide.

## Results

### MOR mediate the deleterious effect of opioids on regenerative process

**Fig. 1:**
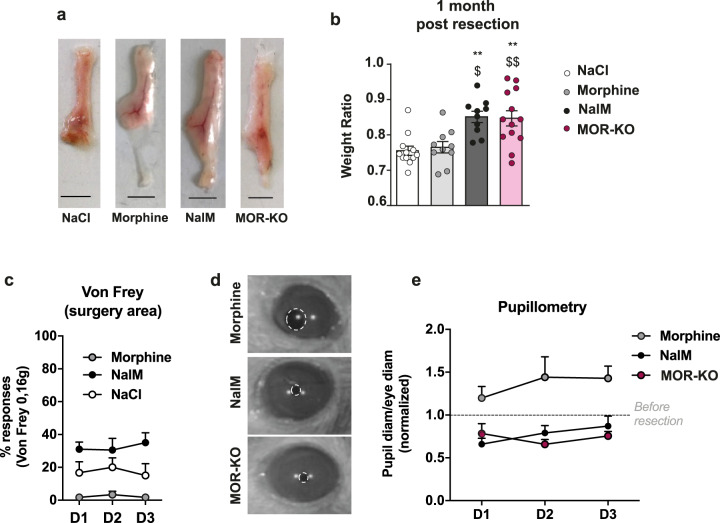
MORs mediate the deleterious effect of opioids on regenerative process. **a** Representative pictures of scAT 1 month after resection, in C57Bl/6 mice treated with NaCl, morphine or NalM, and in MOR-KO mice (scale bars: 0.5 cm). **b** Quantification of regeneration. Weight ratio 1 month post-resection (*n* = 10–13 per group). **c** Quantification of nociceptive sensitivity using von Frey test. Mean frequency (±SEM) of withdrawal reflex after stimulation of the surgery area from day 1 (D1) to day 3 (D3) post-resection (*n* = 6–10 per group). **d** Representative pictures of pupils of C57Bl/6 mice treated with NalM or morphine, and of MOR-KO mice. **e** Quantification of nociceptive sensitivity using pupillary reflex test. Diameter ratio from day 1 (D1) to day 3 (D3) post-resection (*n* = 5–8 per group). NalM: naloxone methiodide. (*^,$^*p* < 0.05, **^,$$^*p* < 0.01. *: vs. NaCl, $: vs. morphine).

After resection in C57Bl/6 mice, scAT heals spontaneously, resulting in the formation of a scar characterized by the absence of differentiated adipocytes and the presence of high-fibrotic collagen deposits^13^. Previously, we demonstrated that the inhibition of opioid receptors using naloxone methiodide (NalM)-induced regeneration of this tissue^13^. The regeneration of scAT was revealed by the increase in weight ratio and the exhibition of adipocytes, blood vessels and nerves organized in a typical shape and structure similar to the ones observed in the contralateral scAT used as an internal control^13^. NalM is a non-selective and competitive antagonist of µ- (MOR), δ- (DOR) and κ- (KOR) opioid receptors that is restricted to peripheral tissues as it does not cross the blood–brain barrier. Given that NalM has greater affinity for MOR than DOR and KOR^14^, we examined the role of MOR in the regenerative process. Morphine also exerts its effects mainly via MOR, so we first confirmed that mice treated with this opioid exhibited scar healing, then we showed that mice treated with NalM regenerated. Indeed, similarly to control (NaCl), morphine-treated mice displayed scar healing of the resected scAT 1 month after resection, while NalM treatment induced macroscopic scAT regeneration (Fig. 1a) which was associated with an increased weight ratio between the resected scAT and its contralateral uninjured counterpart (Fig. 1b). Then, we resected the scAT of mice in which the µ-opioid receptor was genetically ablated (MOR-KO mice) and showed that non-treated MOR-KO mice are able to spontaneously regenerate their scAT 1 month after resection, similarly to wild-type mice treated with NalM (Fig. 1a, b). These data indicate that the deleterious effect of endogenous opioids on regeneration mainly involves MOR.

### Nociception is associated with regeneration in adult mice

Given that peripheral MOR are expressed primarily in nociceptive neurons, we wanted to determine whether regeneration was associated with nociceptive sensitivity. To that aim, we assessed nociception in scar healing and regenerative conditions using a behavioural test exploring mechanical sensitivity (von Frey filaments)^15^. As expected, compared to NaCl, morphine treatment strongly inhibited nociception, assessed by a significant decrease in the percentage of retraction response induced by the application of the 0.16 g von Frey filament on the surgery area (Fig. 1c and Table 1). Moreover, compared to these scar healing conditions (NaCl and morphine), the regenerative condition (NalM) was characterized by a significant increase in the percentage of retraction response (Fig. 1c and Table 1). Same results were obtained using the 0.07 and 1.4 g filaments (Supplementary Fig. 1a, b and Table 1), suggesting that scAT nociceptive sensitivity was greater in regenerative condition. The von Frey filament test was not suitable for testing the sensitivity of MOR-KO mice because of their hypersensitivity to mechanical stimuli in the basal state, outside of any injury context. To overcome this inconvenience, we adapted to mice the pupillary reflex test, a physiological test modulated by nociception^15,16^. We measured the diameters of the pupil and the eye and quantified the nociceptive sensitivity by calculating the ratio between the pupil and the eye diameters. We first controlled that before resection, the diameter ratio was similar between the different groups (Supplementary Fig. 1c, d), then we compared the diameter ratio between these groups after resection. While, as expected, morphine induced statistically significant mydriasis (pupil dilation) after resection, NalM-treated mice and MOR-KO mice displayed significant higher miosis (pupil contraction) compared to control condition (before resection), reflecting an increased nociceptive sensitivity for NalM and MOR-KO groups of animals (Fig. 1d, e and Table 1).

**Table 1:** Statistical information.

Figure	Test	*F* value	*p* Value	
Fig. 1b Weight ratio	One-way ANOVA	*F* (3, 41) = 8,706 *p* = 0.0001	vs. NaCl = *, vs. Morphine = $ NaCl vs. NalM *p* = 0.0025 NaCl vs. MOR-KO *p* = 0.0019	Morphine vs. NalM *p* = 0.0111 Morphine vs. MOR-KO = 0.0097
Fig. 1c Von Frey 0,16	Two-way ANOVA	*F* (2, 57) = 21,45 *p* < 0.0001	Bonferroni post test D1:Morphine vs. D1:NalM *p* = 0.0192 D2:Morphine vs. D2:NalM *p* = 0.0445	D3:Morphine vs. D3:NalM *p* = 0.0038
Fig. 1e Pupillometry	Two-way ANOVA	*F* (2, 51) = 19,44 *p* < 0.0001	Tukey’s post test D2:Morphine vs. D2:NalM *p* = 0.0294 D2:Morphine vs. D2:MOR-KO *p* = 0.0120	
Supp 1a Von Frey 0,07	Two-way ANOVA	*F* (2, 63) = 19,50 *p* < 0.0001	Tukey’s post test D2:Morphine vs. D2:Nal-M *p* = 0.0033 D3:Morphine vs. D3:Nal-M *p* = 0.0242	
Supp 1b Von Frey 1,4	Two-way ANOVA	*F* (2, 63) = 35,03 *p* < 0.0001	Bonferroni post test D1:Morphine vs. D1:Nal-M *p* = 0.0005 D2:Morphine vs. D2:Nal-M *p* = 0.0004	D3:Morphine vs. D3:Nal-M *p* = 0.0002
Fig. 2b Nerve fibres	*t* Test		**p = 0.0026	
Fig. 2e Von Frey 0,16	Two-way ANOVA	*F* (1, 33) = 25,78 *p* < 0.0001	Bonferroni post test D1:Nal-M vs. D1:Capsaicin + Nal-M *p* = 0.0033	
Fig. 2h Weight ratio	*t* Test		***p = 0.0008	
Fig. 3b Weight ratio	*t* Test		**p = 0.0059	
Fig. 3d Weight ratio	One-way ANOVA	*F* (2, 12) = 6,082 *p* < 0.015	Morphine vs. CGRP *p* = 0.0379 Morphine vs. Morphine + CGRP *p* = 0.0199	
Fig. 3e Von Frey 0,16	Two-way ANOVA	*F* (2, 63) = 94,16 *p* < 0.0001	Bonferroni post test D1:Morphine vs. D1:CGRP *p* < 0.0001 D2:Morphine vs. D2:CGRP *p* < 0.0001 D3:Morphine vs. D3:CGRP *p* < 0.0001	D1:CGRP vs. D1:Morphine + CGRP *p* < 0.0001 D2:CGRP vs. D2:Morphine + CGRP *p* < 0.0001 D3:CGRP vs. D3:Morphine + CGRP *p* < 0.0001

Thus, mice of which MOR are blocked or disabled, are able to regenerate their scAT and exhibit increased nociceptive sensitivity suggesting that nociceptive nerves could be involved in regenerative process.

### Selective denervation of nociceptive neurons impairs tissue regeneration

**Fig. 2:**
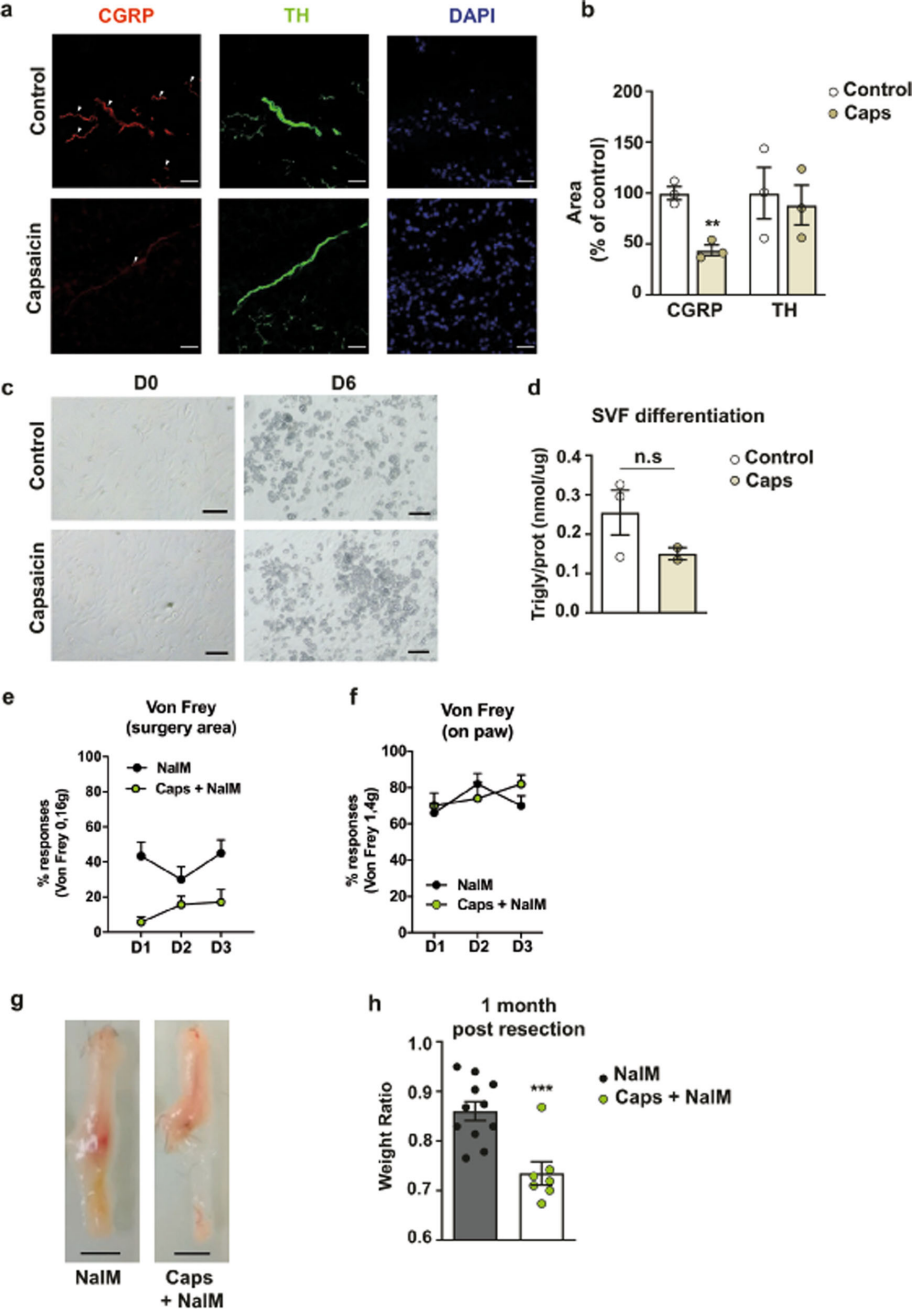
Selective denervation of nociceptive sensory neurons impairs tissue regeneration. **a** Imaging of control or denervated scAT, 21 days after capsaicin injection, showing sensory fibres (CGRP, red), sympathetic fibres (TH, green) and nuclei (DAPI, blue) (scale bars: 0.5 cm). **b** Quantification of sensory (CGRP) and sympathetic fibres (TH) (*n* = 3). **c** Representative pictures of the stromal vascular fraction isolated from scAT 21 days after injection of capsaicin and placed in differentiation medium for 6 days (scale bars: 100 µm). **d** Quantification of triglycerides related to total proteins (nmol/µg) after 6 days of culture in differentiation medium (*n* = 2–3 per group). **e**, **f** Quantification of nociceptive sensitivity using von Frey test. Mean frequency (±SEM) of withdrawal reflex after stimulation of the surgery area (**e**) (*n* = 6–7 per group) or the paw (**f**) (*n* = 6–7 per group), from day 1 (D1) to day 3 (D3) post-resection, in mice treated with NalM and previously denervated with capsaicin or not. **g** Representative pictures of scAT 1 month after resection and morphine or NalM treatment in mice previously denervated with capsaicin or not (scale bar: 0.5 cm). **h** Quantification of regeneration. Weight ratio 1 month post-resection (*n* = 7–11 per group). CGRP: calcitonin gene-related peptide, TH: tyrosine hydroxylase, NalM: naloxone methiodide, Caps: capsaicin. (**p < 0.01, ***p < 0.001).

In order to determine the role of nociceptive neurons in tissue regeneration, we have specifically disrupted them using high doses of capsaicin. Capsaicin, the compound of hot chilli peppers, is a ligand of the TRPV1 cation channel and its expression is restricted to peptidergic nociceptive neurons^17^. Upon its activation by capsaicin, TRPV1 channel opens, leading to an influx of calcium and sodium ions into the neuron and triggering its depolarization. At normal levels, capsaicin binding transmits the sensation of pain. However, high doses of capsaicin lead to a massive influx of ions, resulting in the death of neurons expressing TRPV1^18^. As expected, 21 days after capsaicin treatment, immunostaining against the sensory neuropeptide CGRP showed a clear reduction of CGRP innervation compared to control, while the number of sympathetic autonomous (tyrosine hydroxylase, TH^+^) fibres was unaffected (Fig. 2a, b). Thus, denervation was effective and specifically targeted the scAT sensory but not sympathetic nerves. To identify a putative effect of such denervation on adipose tissue features, we carefully examined the cell heterogeneity of capsaicin-treated tissue. The density of adipocytes as well as the number of adipocyte progenitors (adipose stromal/stem cells, ASCs), endothelial and immune cells in the stromal vascular fraction has not changed 21 days after denervation suggesting that such denervation did not affect the tissue biology (Supplementary Fig. 2a–e). Furthermore, the similarity in the number of macrophages CD45^+^/F4/80^+^/CD11b^+^ (Supplementary Fig. 2c, f) between both groups suggested that denervation did not induce prolonged inflammation of the scAT. Lastly, the ability of ASCs of capsaicin-treated mice to differentiate into adipocytes showed no significant difference with control ASCs, indicating that the ability of ASCs to differentiate into adipocytes was not altered by capsaicin treatment (Fig. 2c, d). Altogether, these results demonstrate that denervation of nociceptive fibres does not alter the biology of the scAT. After NalM treatment of resected mice, nociceptive sensitivity was significantly decreased by denervation as showed by the decrease of the percentage of retraction response to von Frey filament stimulation of the surgery area in capsaicin-treated mice (Fig. 2e and Table 1). This confirmed the efficiency of the denervation. In contrast, paw sensitivity was unaffected by scAT denervation (Fig. 2f and Table 1), indicating that denervation with capsaicin effectively altered the nociceptive sensitivity only of the scAT. Then, we examined the regeneration capability of both groups 1 month after resection. We showed that in contrast to NalM mice that exhibited macroscopic scAT regeneration, mice that were denervated with capsaicin prior to resection and treatment with NalM showed scar healing (Fig. 2g). This was associated with a significant reduction in the weight ratio between the resected scAT and its contralateral uninjured counterpart (Fig. 2h).

These results show that selective denervation of nociceptive neurons in scAT impairs tissue regeneration in adult mice and highlight the involvement of nociceptive neuron signalling in the regenerative process.

### CGRP is sufficient to induce regeneration of the injured tissue and overcomes opioid scar healing effect

**Fig. 3:**
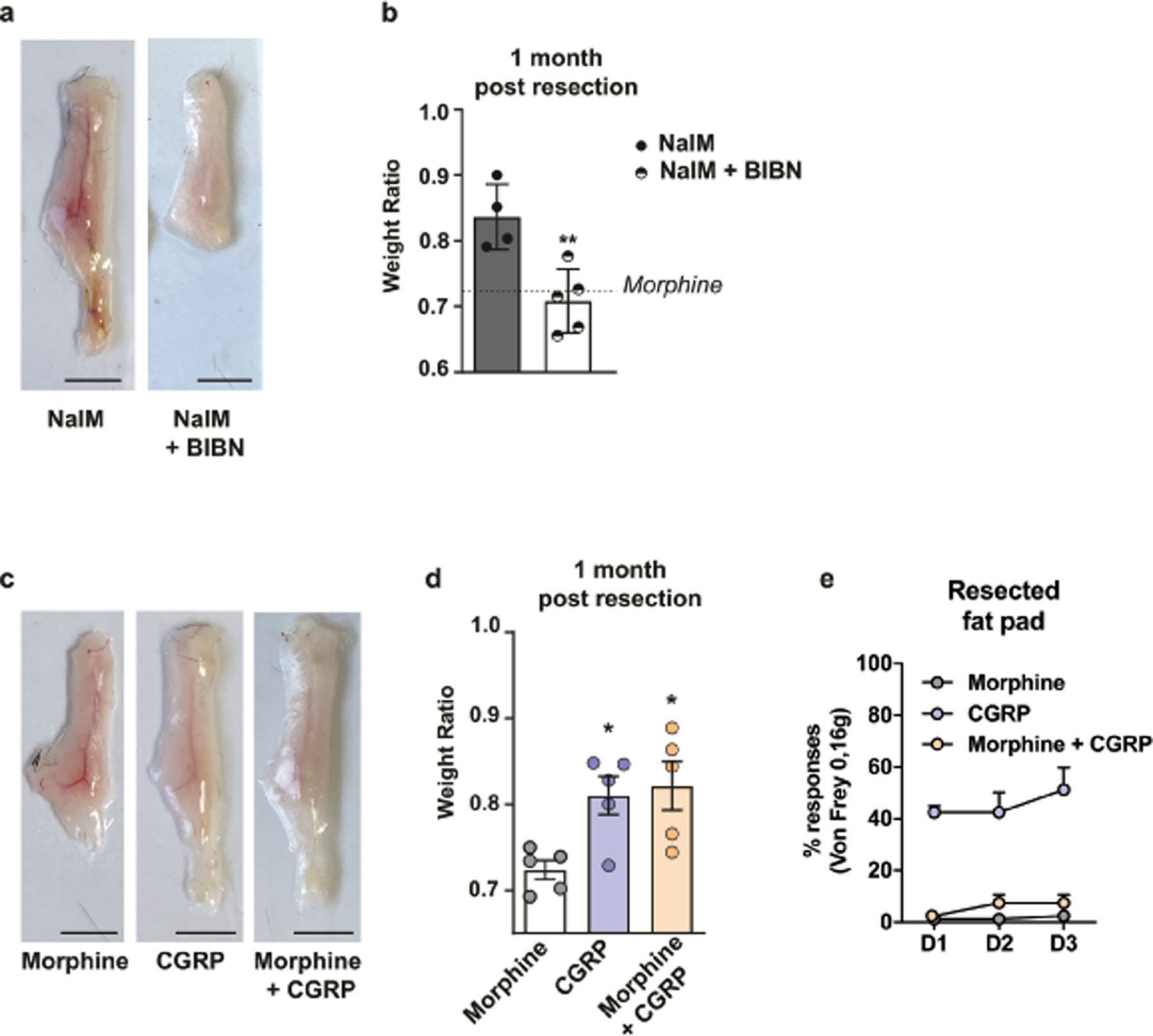
CGRP is sufficient to induce regeneration of the injured tissue and overcomes opioids’ scar healing effect. **a** Representative pictures of scAT, 1 month after resection and NalM or NalM + BIBN treatment (scale bars: 0.5 cm). **b** Weight ratio quantification (*n* = 4–5 per group). The dotted line indicates the weight ratio in scar healing condition (morphine). **c** Representative pictures of scAT, 1 month after resection and morphine or CGRP or morphine + CGRP treatment (scale bars: 0.5 cm). **d** Weight ratio quantification (*n* = 5 per group). The dotted line indicates the weight ratio in scar healing condition (morphine). **e** Quantification of nociceptive sensitivity using von Frey test. Mean frequency (±SEM) of withdrawal reflex after stimulation of the surgery area from day 1 (D1) to day 3 (D3) post-resection (*n* = 5 per group). NalM: naloxone methiodide, BIBN: CGRP receptors antagonist BIBN-4096, CGRP: calcitonin gene-related peptide. (*p < 0.05, **p < 0.01).

The analgesic effect of opioid drugs is mediated by reducing the release of neuropeptides such as CGRP from the central and peripheral terminals of nociceptive neurons^10^. To test whether the nociceptive nerve-dependent regeneration of scAT relies on the neuropeptide CGRP signalling, we induced regeneration with NalM in scAT-resected mice and injected them subcutaneously with the selective CGRP receptor antagonist BIBN^19^. As expected, only mice treated with NalM regenerated. In contrast, mice treated with NalM and BIBN displayed macroscopic scar healing 1 month after resection (Fig. 3a) and a significant decrease in the scAT weight ratio (Fig. 3b). These data suggested that CGRP was required for NalM-induced scAT regeneration in adult mice. To determine whether CGRP was sufficient to induce regeneration independently of the NalM treatment, we performed scAT resection and treated the mice only with CGRP by subcutaneous injections for 3 days after injury. In contrast to the morphine-treated mice, the CGRP-treated mice exhibited macroscopic scAT regeneration 1 month after resection (Fig. 3c) and quantification of regeneration showed a significant increased weight ratio following CGRP treatment (Fig. 3d).

Since the management of severe acute nociceptive pain is an ethical obligation, the challenge is to induce tissue regeneration while providing analgesia. Taking advantage of the CGRP pro-regenerative properties, we administrated CGRP locally in combination with a systemic injection of morphine to the resected mice to provide pain management. The morphine + CGRP-treated mice demonstrated macroscopic regeneration of scAT 1 month after resection (Fig. 3c) and their weight ratio was similar to the CGRP mice (Fig. 3d), suggesting that CGRP induced regeneration despite morphine treatment. Thus, CGRP treatment counteracted the scar healing effect of morphine. To determine whether the co-administration of CGRP and morphine affected the analgesic effect of morphine, we evaluated the nociceptive sensitivity of mice. The morphine + CGRP mice demonstrated a significant decrease in the percentage of retraction response to von Frey filament stimulation when compared to CGRP mice (Fig. 3e and Table 1). Their percentage of retraction reached the value of morphine mice, suggesting that morphine maintained its analgesic effects despite CGRP treatment (Fig. 3e). Thus, morphine treatment counteracted the general pro-nociceptive effect of CGRP with no effect on its local pro-regenerative properties.

Altogether, these results show that co-administration of morphine with CGRP after resection induces tissue regeneration while providing analgesia.

## Discussion

Numerous reports across multiple species suggest that innervation is essential for appendage regeneration^2,4,6^; but the nature of this innervation remains largely undefined. In this study, using pharmacological approaches together with behavioural and reflex tests, we demonstrate that nociceptive nerves are essential for tissue regeneration in adult mice and are inhibited by endogenous opioids.

We previously demonstrated that endogenous and exogenous opioids impair spontaneous regenerative process in adult vertebrates^13^. Opioid effects are mediated by several receptors^20^ and we show here that anti-regenerative effects of opioids are mediated at least in part by MOR. Consistent with our conclusion, a recent study in a model of acute pancreatitis, demonstrated that epithelial pancreatic regeneration is delayed by morphine treatment via MOR^21^, due to a delay in macrophage recruitment in the tissue of mice receiving morphine. According to the close link between opioids and nociception and the fact that MOR are mainly expressed by nociceptive neurons in peripheral tissue, we investigated the role of nociceptive neurons in the regenerative process through their selective depletion using the neurotoxin capsaicin^17^. The impairment of the regeneration induced by the selective loss of these neurons clearly shows that nociceptive innervation is required for regenerative process. An abundance of literature is consistent with this conclusion. A recent study restricted to the role of the TRPA1 cation channels identified that pharmacological activation of these channels promotes mouse skin regeneration^22^. Because these channels are expressed on peripheral sensory neurons this could indirectly suggest that tissue regeneration can be induced by stimulation of sensory neurons. The lack of nociceptive innervation was reported to negatively affect the repair of cutaneous and corneal wounds after moderate lesion^23,24^. Finally, our conclusion on the pro-regenerative effect of nociceptive neurons could be shared by other vertebrates than mammals such as lower vertebrates, as pointed out by the study from Meda et al.^25^ showing the role of sensory innervation in the regeneration of the caudal fin in zebrafish.

The alteration of nociceptive neurons using capsaicin prevented their activation and neuropeptide release^26^ and revealed the role of the nociceptive neuropeptide in regenerative process. Indeed, disruption of CGRP signalling by pharmacological approaches affected the ability to regenerate, whereas treatment of mice with CGRP following resection (i) was sufficient to induce regeneration of damaged tissue, and (ii) counteracted the scar healing effect of morphine. Altogether, though we cannot rule out the intervention of other neuropeptides, our pharmacological loss- and gain-of-function approach targeting the signalling of the sensory neuropeptide CGRP suggests that nociceptive neurons control regeneration probably through the release of CGRP. The local role of CGRP on acceleration of wound closure after skin injury was previously reported without being known whether this effect was a pro-regenerative or a pro-scar healing effect^27,28^. In our scAT regenerative model, CGRP could target several specific cell types. Indeed, CGRP has been described to interact with adipocytes, lowering energy utilization^29^, to promote the migration of umbilical cord mesenchymal stem cells to the injury site of transected spinal cord^30^, to act directly on smooth muscle cells to promote vasodilation, favouring the recruitment of immune cells, and to bind on macrophages and dendritic cells, affecting cytokine production^31^. We have preliminary data indicating that ASCs express all three proteins that make up the CGRP receptor, however, the cell types with which nociceptive neurons interact remains to be investigated.

A great originality of our work is to investigate the link between tissue regeneration and nociception. We performed two types of measures, the von Frey and the pupillary dilatation reflex test. Though the behavioural Von Frey test is the gold-standard test to estimate nociception, it cannot be used in hypersensitive models such as MOR-KO mice. As an alternative, the reflex pupillary test can be used. It is a supraspinal reflex in response to noxious stimulation that is routinely performed using portable infrared pupillometry test in the clinic to monitor analgesia during surgery in anaesthetized patients^32–34^. Pupil diameter is under dual sympathetic/parasympathetic control, the sympathetic (noradrenergic) output dilating the pupil, and the parasympathetic (cholinergic) output constricting the pupil. In diurnal animals (rabbit, man) noxious stimuli may activate predominantly the sympathetic premotor neurones that receive nociceptive inputs, whereas in nocturnal animals (mice, rat, cat) they may activate predominantly the parasympathetic premotor neurones. Thus in mice, noxious stimulation results in pupil contraction (miosis) which is suppressed by the administration of analgesic drugs^15^. Though the involvement of nociceptive neurons was clearly demonstrated using both tests, the requirement of autonomous nerve fibres in sensory–motor loops cannot be ruled out. Interestingly, the critical role of cholinergic nerves for heart regeneration has been reported in zebrafish and neonatal mice^35^. Though scAT lacks parasympathetic innervation, the presence of sympathetic innervation is well established^36^ and it remains to be determined whether these nerve fibres also control scAT regeneration.

Our previous report and the present work using pharmacological (NalM) and genetic (MOR-KO) inhibition of opioid receptors reveal an endogenous potential for adult mammals’ tissue to regenerate, which is triggered by spontaneous activation of nociceptive neurons after injury. This means that nociceptive neurons would have two distinct and complementary functions after injury (i) to protect the organism by activating protective physiological and behavioural mechanisms, and (ii) to participate to recover functional tissue by controlling repair processes. Our data also reveal that, in adult mammals, activation of the endogenous opioid system following lesion inhibits the regenerative process induced by nociceptive neurons. These opposite effects of nociceptive system and opioids on repair processes, i.e. regenerative or scar healing, could suggest that the repair outcomes observed in different animals and situations could result from the balance between them.

Throughout their lives, mammals experience changes in regenerative capacity of many physiological systems, including heart, spinal cord, digit tips and hair cells. For example, their ability to regenerate their hearts is lost shortly after birth^37^. We can propose that age differences in nociceptive pathways and the opioid system may be involved in the variation in regenerative capacity observed through ontogeny and aging. At early postnatal ages, noxious stimulation mainly results in a prolonged electrical activity that lasts beyond the end of the stimulus^38^. This exaggerated and sometimes inappropriate response to noxious stimuli disappears with the maturation of the nociceptive circuitry in parallel with a more efficient activity of the dorsal horn and descending inhibitory pathways^39^. In addition to the reorganization of the spinal connectivity, the endogenous opioid system also undergoes a postnatal maturation^40,41^. The MOR receptors are present at birth but their binding to opioids may not necessarily be associated with intracellular signalling activation until postnatal development^42^. This finding may account for the lower analgesic potency of morphine on noxious thermal stimulation that is observed at birth^43^. With advanced age, the already limited regenerative capacity of adult mammals is further reduced; regenerative organs and tissues including blood, muscle and bone, experience degeneration and dysfunction^44^. Interestingly, if there is a general decline in the opioid system with age^45^, functional, structural, and biochemical changes have also been reported in peripheral nerves of aged subjects, with a marked reduction in the density of nociceptive fibres and nociceptive peptide content as well as an increased number of sensory fibres with signs of damage or degeneration^46^. Although this demonstration will require fine assessment of these elements in different models and at different stages of development, these data suggest that there is an association between regenerative capacity and the activity of the nociceptive and opioid systems. A recent study shows full-thickness tissue regeneration in an aged murine model of ear hole closure, it would be interesting to investigate the nociceptive innervation in this model^47^.

It is interesting to note that NalM or CGRP are transiently administrated during 4 days after the lesion and the mice do not receive any other treatment until the regeneration is evaluated 1 month later. Thus, early and transient treatment after the injury triggers lasting long-term effects that lead to tissue regeneration. This suggests that activation of nociceptive nerves may be the starting point for successive events that lead to the replacement of the tissue. Lastly, because local administration of the sensory neuropeptide CGRP is sufficient to induce regeneration of damaged tissue and overcome the scar healing effect of morphine while allowing for general analgesia, we are therefore able to propose a new post-operative analgesic strategy that promotes tissue regeneration through the morphine/CGRP co-administration. A body of works links sensory neuropeptides to the formation of heterotopic ossification in soft tissues following traumatic injury, and locations of heterotopic ossification are generally associated to the presence of stromal cells with osteogenic potential^48^. Upon activation by BMP2 delivery in the muscle, sensory neurons induce neurogenic inflammation, resulting in the migration of osteogenic stem cells from the nerve^49^. However, it was demonstrated that if administration of substance P alone promotes heterotopic ossification formation with increased expression of BMP2, CGRP alone has no significant effect. Remarkably, when delivered with substance P, CGRP counteracts substance P-induced heterotopic ossification^50^. In addition to the fact that in our model we did not observe any heterotopic ossification in the regenerated adipose tissue, these data therefore support our proposed strategy of using CGRP to promote tissue regeneration, without fear of inducing heterotopic ossification.

All together, these results open new perspective on the link between nociception and regeneration in adult mammals and questions about the putative effects of the post-operative analgesic care on repair processes. Fortunately, it seems that new post-operative pro-regenerative analgesic strategy can be proposed from the deciphering of underlined local mechanisms as we demonstrated with a morphine/CGRP co-administration.

## Methods

### Animals

All experiments were performed on 5- to 7-week-old male mice. C57BL/6 mice were obtained from Harlan Laboratories and bred in the CREFRE (Centre Regional d’Exploration Fonctionnelle et Ressources Expérimentales) and MOR-KO mice were kindly provided by G. Mithieux. Animals were group-housed (3 or 4 per cage) in a controlled environment (12-hour light/dark cycle at 21 °C) with unrestricted access to water and a standard chow diet in a pathogen-free animal facility. The animals were maintained in accordance with the guidelines of the European Community Council. Mice were killed by cervical dislocation. All experiments were carried out in compliance with European Community Guidelines (2010/63/UE) and approved by the French ethics committee (protocol reference: 2016031009332865).

### In vivo treatments

Mice were treated with naloxone methiodide (NAL-M) (subcutaneous injection, 17 mg/kg, N129, Sigma Aldrich, Saint Louis, MO, USA), morphine (subcutaneous injection, 10 mg/kg, Francopia, Antony, France), CGRP (subcutaneous injection, 7.5 µg/mice, 1161/100U, R&D Systems, Minneapolis, MN), or the CGRP receptor antagonist BIBN4096 (subcutaneous injection, 1 mg/kg, Cat. No. 4561, R&D Systems, Minneapolis, MN) on days 0–3 after scAT resection.

### scAT resection

Control mice were used for the baseline control and did not undergo surgery. Mice underwent unilateral resection of scAT as described in our previous report^13^. Briefly, animals were anaesthetized by inhalation of isoflurane 2.5% and a single incision was made on the abdomen to access and excise 35% of the right scAT between lymph node and groin. The left scAT did not undergo surgical procedure and was used as an internal control. To quantify scAT regeneration, the weight ratio between the right (i.e. resected) and the left (i.e. contralateral) scAT was calculated^13^.

### scAT denervation

scAT sensory denervation was adapted from a protocol previously published by Vaughan et al.^51^. Capsaicin (M2028, Sigma Aldrich, St Louis, MO, USA) was diluted in 100% ethanol, then in olive oil (1:10, 01514, Sigma Aldrich, St Louis, MO). Animals were anesthetized by inhalation of isoflurane 2.5% and scAT was wetted with NaCl 0.9% during the length of experiment. Briefly, 8 micro injections of 5 µL capsaicin (20 µg/µL) were performed in scAT using Hamilton needle (Dutsher, 25 µL).

### Behavioural and reflex tests

#### von Frey

Mechanical nociceptive sensitivity (allodynia) was quantified by measuring the withdrawal response to von Frey filament stimulation. Unrestrained mice were placed beneath a clear plastic chamber on an elevated mesh floor and were allowed to settle for 45 min. Withdrawal responses to mechanical stimulation were determined using calibrated von Frey filaments applied from underneath the cage through the mesh floor to the scAT surgery area or to the hind paw plantar skin on the same side as the resection. Testing was carried out with von Frey filaments of increasing stiffness (from 0.07 to 1.4 g). Each trial consisted of 10 applications of the filament. The mouse reaction to von Frey filament was scored as percentage of withdrawal.

#### Pupillometry

Mice were briefly anesthetized (40 s, 1–2% isoflurane) and a picture of the open eye was taken using an infrared portative camera equipped with a macro lens (Neurolight^®^ device, IDMED). The nociceptive sensitivity was quantified by calculating the ratio between the diameter of the pupil and the eye. Mydriasis reflects low nociceptive sensitivity while miosis reflects increased nociceptive sensitivity.

### Immunohistochemistry

First, 300 µm scAT sections were incubated in blocking solution (2% Normal Horse Serum and 0.2% triton X-100 in PBS) then with CGRP (rabbit, 1:350, PC205L, Sigma Aldrich, St Louis, MO) and tyrosine hydroxylase (TH) (sheep, 1:750, AB1542, Sigma Aldrich, St Louis, MO) antibodies at room temperature for 24 h. Then sections were incubated overnight at 4 °C with Al594 donkey anti-rabbit (1:250, Molecular Probes A21207) and Al488 donkey anti-sheep (1:250, Molecular Probes A11015) secondary antibodies, mounted on a coverslip and imaged using a confocal laser scanning microscope (LSM780, Carl Zeiss, Oberkochen, Germany). Images were processed using Fiji software (NIH, Bethesda, MD, USA).

### Statistical analyses

Studies were not randomized and investigators were blinded to analyses. All results are given as mean ± SEM. Data were analysed using *t* test or a two-way ANOVA for von Frey and pupillometry tests and a one-way ANOVA for weight ratio quantification (Table 1). All statistical analyses were performed in GraphPad Prism 9.1.2 software and a two-tailed *p* value with 95% confidence interval was acquired. The results of the statistical analyses are summarized in Table 1.

### Reporting summary

Further information on research design is available in the Nature Research Reporting Summary linked to this article.

## Supplementary information

Supplementary information.

Reporting summary.

## Data Availability

The data that support the findings of this study are available from the corresponding author upon reasonable request.
