# Supplementary material for: Pain sensing neurons promote tissue regeneration in adult mice
Source: NPJ Regen Med. 2021 Oct 14;6:63. doi: 10.1038/s41536-021-00175-7 (PMC8516997; doi:10.1038/s41536-021-00175-7)
Supplement: Supplementary file 1 — Supplementary information. [file 41536_2021_175_MOESM1_ESM.pdf]

Supplementary Figure 1

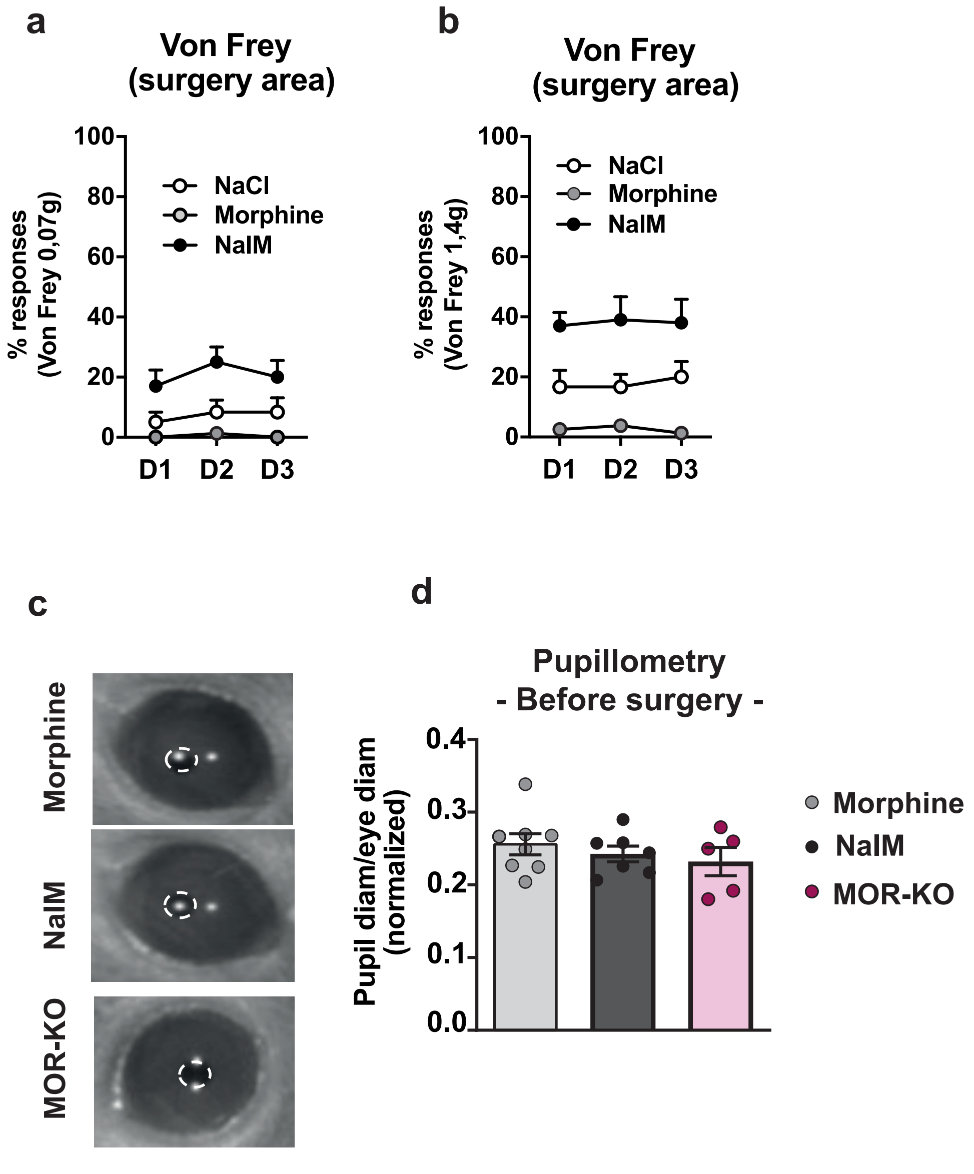

Supplementary Figure 1.
(a,b) Quantification of nociceptive sensitivity using von Frey test. Mean frequency (± SEM) of withdrawal reflex after stimulation of the surgery area with the 0,07g filament (a) and the 1,4g filament (b) from day 1 (D1) to day 3 (D3) post-resection in NaCl, morphine or NalM-treated mice (n=6-10 per group). (c) Representative pictures of pupil before resection. (d) Quantification of nociceptive sensitivity using pupillary reflex test before surgery (n=5-8 per group). NalM: Naloxone Methiodide.

Supplementary Figure 2

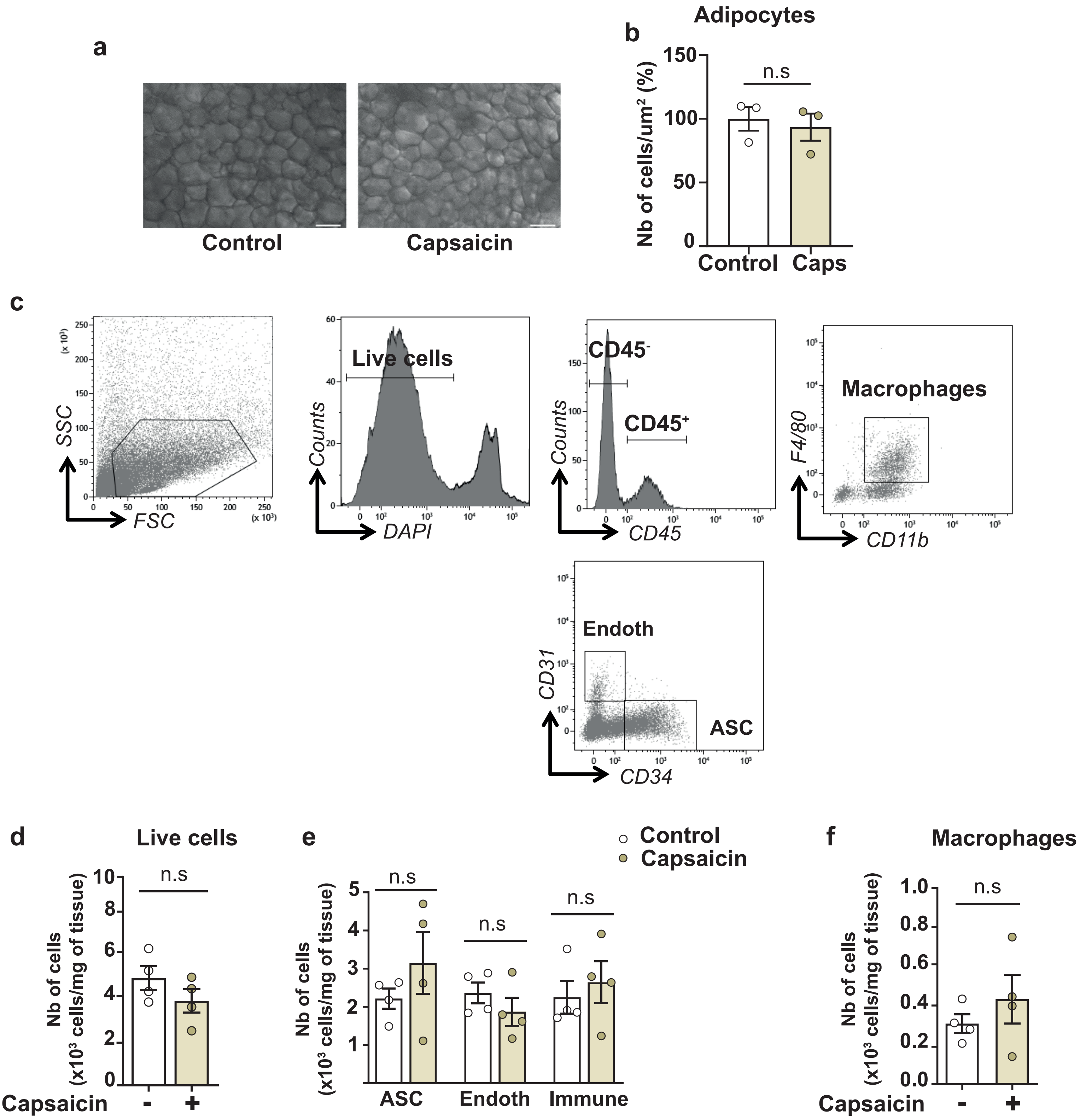

Supplementary Figure 2.

(a) Representative pictures of scAT, 21 days after capsaicin injection (scale bars: 50µm). (b) Quantification of adipocyte density in the capsaicin-treated area (n=3 per group). (c) Gating strategy: Cells were first gated on FSC/SSC to eliminate debris, before gating on DAPI to identify Live cells. F4/80 and CD11b staining was then analysed on $CD45^+$ (immune) cells to identify macrophages. ASC and endothelial cells were identified in $CD45^-$ cells as $CD34^+/CD31^-$ and $CD34^-/CD31^+$ respectively. To accurately define positive versus negative cells for each antibody, isotype controls were used. (d) Quantification of Live cells in scAT, gated on DAPI negative cells by flow cytometry 21 days after capsaicin injection. (e) Quantification of Adipose Stromal Cells (ASC) gated on $CD45^-/CD31^-/CD34^+$ cells, endothelial cells gated on $CD45^-/CD31^+/CD34^+$ cells and immune cells gated on $CD45^+/CD31^-/CD34^-$ cells, in scAT, 21 days after capsaicin injection (n=4 per group). (f) Quantification of macrophages ($CD45^+/CD11b^+/F4/80^+$) in scAT, 21 days after capsaicin injection (n=4 per group). Caps: capsaicin, ASC: Adipose Stromal Cells, Endoth: endothelial cells.
